# The Effect of Oxygenation on Mortality in Patients With Head Injury

**DOI:** 10.7759/cureus.34385

**Published:** 2023-01-30

**Authors:** Zehra Kılınç, Elif Aybike Ayyıldız, Ebru Kaya, Ayca Sultan Sahin

**Affiliations:** 1 Anesthesiology and Reanimation, Kanuni Sultan Süleyman Training and Research Hospital, Istanbul, TUR; 2 Intensive Care Unit, Kanuni Sultan Süleyman Training and Research Hospital, Istanbul, TUR

**Keywords:** hypoxic brain injury, head trauma, cerebral oxygenation, intensive care medicine, hyperoxygenation

## Abstract

Introduction

In this study, we planned to investigate the effect of hyperoxygenation on mortality and morbidity in patients with head trauma who were followed and treated in the intensive care unit (ICU).

Methods

Head trauma cases (n = 119) that were followed in the mixed ICU of a 50-bed tertiary care center in Istanbul between January 2018 and December 2019 were retrospectively analyzed for the negative effects of hyperoxia. Age, gender, height/weight, additional diseases, medications used, ICU indication, Glasgow Coma Scale score recorded during ICU follow-up, Acute Physiology and Chronic Health Evaluation (APACHE) II score, length of hospital/ICU stay, the presence of complications, number of reoperations, length of intubation, and the patient's discharge or death status were evaluated. The patients were divided into three groups according to the highest partial pressure of oxygen (PaO_2_) value (200 mmHg) in the arterial blood gas (ABG) taken on the first day of admission to the ICU, and ABGs on the day of ICU admission and discharge were compared.

Results

In comparison, the first arterial oxygen saturation and initial PaO_2_ mean values were found to be statistically significantly different. There was a statistically significant difference in mortality and reoperation rates between groups. The mortality was higher in groups 2 and 3, and the rate of reoperation was higher in group 1.

Conclusion

In our study, mortality was found to be high in groups 2 and 3, which we considered hyperoxic. In this study, we tried to draw attention to the negative effects of common and easily administered oxygen therapy on mortality and morbidity in ICU patients.

## Introduction

Trauma is one of the most important causes of death in young adults in our country and worldwide. Head trauma is present in a significant percentage of trauma patients. Mortality is higher in these cases. Follow-up and treatment for most of these patients are carried out in the intensive care unit (ICU) [1,2].

When oxygen is used in the long term and in high concentrations, oxidants and free radicals that cause irreversible cell injury are formed. This injury gradually increases beyond a certain concentration. Therefore, oxygen therapy should be applied as short as possible and at the lowest concentration. The indication and extent of oxygen therapy are determined by oxygen saturation measurements with non-invasive and invasive methods. Additional measures are also evaluated, including arterial blood gas (ABG) analysis, pulse oximetry, respiration rate and pattern, use of accessory muscles, and pulse and blood pressure. Especially, partial pressure of oxygen (PaO_2_), assessed with ABG, is very important for the initiation and continuation of oxygen therapy [3,4].

Most of the studies about hyperoxygenation performed in ICUs are mostly retrospective and include very heterogenic groups. This might be the main reason for insignificant results. The results of most of these studies show that hyperoxygenation is harmful [5].

In most patients, oxygen therapy is performed without any information about tissue oxygenation. Yet, it is a supportive treatment, which does not have a very high therapeutic window. It should be kept in mind that uncontrolled oxygen therapy can cause irreversible cell damage by producing toxic oxygen metabolites such as superoxide anion, hydrogen peroxide, hydroxyl radicals, and singlet oxygen [6,7].

In this study, the effect of hyperoxygenation on mortality and morbidity in patients with head trauma who stayed in the ICU is investigated by comparison of blood gas samples that are obtained on the first and the last day of ICU stay. This study aims to be useful in decreasing mortality and morbidity, length of hospital stay (LOS), and economic or physical damage that make patient care difficult.

## Materials and methods

Data from patients with head trauma who were hospitalized in tertiary ICU between January 2018 and December 2019 were analyzed retrospectively. İstanbul Health Sciences University Kanuni Sultan Süleyman Training and Research Hospital ethical committee approved the study with the decision number KAEK/2018.6.20. A total of 195 patients with head trauma who were admitted to the ICU from other services or other centers were included in our study and evaluated from our hospital database.

Patients who were under 18 years of age (20), had a hospital stay of fewer than 24 hours (39), had chronic lung disease or acute lung injury, and whose files could not be found or had files with missing data (17) were excluded from the study.

Age, gender, height/weight, comorbidities of the patients, drug history, and the unit before ICU admission were recorded. Glasgow Coma Scale (GCS) score, Acute Physiology and Chronic Health Evaluation (APACHE) II score, indications for ICU admission, LOS, number of reoperations, and discharge or death status of the patients were also recorded. GCS scores were recorded on the day of admission and discharge. The values of pH, arterial partial pressure of carbon dioxide (PaCO_2_), arterial PaO_2_, bicarbonate (HCO3), base excess (BE), lactate, and arterial oxygen saturation (SaO_2_) in ABG, which were measured at the first and last day of ICU stay, were recorded. The patients were divided into three groups according to the highest PaO_2_ value in the ABG samples taken on the first day of the ICU. Initial and last ABG values were compared.

Statistical analysis

IBM SPSS Statistics 22.0 program (IBM Corp., Armonk, NY) was used for statistical analysis in our study. When the power analysis was performed, the number of subjects required for each group was calculated to be at least 22. To evaluate demographic and clinical data of the head trauma patients, descriptive analyses like mean, median, minimum, and maximum were used. Kruskal-Wallis H test was used for comparison of the mean values of the three groups, which were created based on initial PaO_2_ values. To analyze categorical variables, the chi-square test was used. When categorical variables were under 5%, Fisher’s exact test was used. Mean values of cases with mortality and without mortality were compared according to the Mann-Whitney U test. The significance level was set at 0.05 for all analyses.

## Results

In our study, we found that the age of the 119 ICU patients ranged from 18 to 91 years. Of the cases, 100 (84%) were males and 19 (16%) were females. The highest initial PaO_2_ values of patients were evaluated and 11 (9.2%) of these were found to be between 0 and 120 mmHg, 88 (73.9%) were between 120 and 200 mmHg, and 20 (16.8%) were above 200 mmHg, respectively. Therefore, the patients included in the study were divided into three groups according to their initial PaO_2_ values (Table 1).

**Table 1:** Comparison of demographic characteristics of patients according to PaO2 values a = Kruskal-Wallis H test; b = Pearson's chi-square analysis; Med. = median; Min = minimum; Max = maximum; BMI = body mass index; PaO_2 _= partial pressure of oxygen.

		PaO_2_									
		0-120 mmHg			120-200 mmHg			>200 mmHg			
		n = 11			n = 88			n = 20			
		Mean	Med.	Min-Max	Mean	Med.	Min-Max	Mean	Med.	Min-Max	p
Age		45	42	28.00-81.00	40.59	36	18.00-91.00	39.25	27	18.00-87.00	0.398^a^
BMI		30.5	32	16.00-46.00	31.98	32	13.00-52.00	29.28	28	19.00-44.00	0.163^a^
		n	%		n	%		n	%		
Gender	Male	7	63.6		77	87.5		16	80		0.109^b^
	Female	4	36.4		11	12.5		4	20		0.109^b^

In comparison, it was found that the mean age (p = 0.398), gender ratios (p = 0.109), and BMI (p = 0.163) were not statistically significant between the three groups.

There was epidural bleeding (EB) in 61 (51.3%), EB + subarachnoid hemorrhage (SAH) in two (1.7%), epidural + subdural bleeding in one (0.8%), SAH in 26 (21%, 8), subdural bleeding in 24 (20.2%), subdural + EB in three (2.5%), and traumatic SAH in two (1.7%) of the cases. In addition, according to the American Society of Anesthesiologists (ASA) classification, 83 (69.7%) patients were found to be grade I, 29 (24.4%) were grade II, and seven (5.9%) were grade III, respectively. The diagnosis rates (p = 0.963) and ASA ratios (p = 0.136) were not statistically different between the three groups.

In the study, 36 (30.3%) patients had comorbidities and the incidence of comorbid diseases was not significantly different (p = 0.060) between groups.

GCS (p = 0.091) and APACHE (p = 0.891) scores were not significantly different between the groups. However, GCS scores were lower in hyperoxic groups 2 and 3 (Table 2).

**Table 2:** Comparison of GCS and APACHE scores according to PaO2 values a = Kruskal-Wallis H test; Med. = median; Min = minimum; Max = maximum; GCS = Glasgow Coma Scale; APACHE = Acute Physiology and Chronic Health Evaluation score; PaO_2 _= arterial partial pressure of oxygen (mmHg).

	PaO_2_												
	0-120 (n = 11)				120-200 (n = 88)				>200 (n = 20)				
	Mean	Med.	Min	Max	Mean	Med.	Min	Max	Mean	Med.	Min	Max	p^a^
GCS	9.45	10.00	3.0	15.00	8.52	9.00	3.00	14.00	6.95	8.00	3.00	15.00	0.091
APACHE	19.55	18.00	12.00	40.00	18.07	17.00	10.00	45.00	19.25	16.50	11.00	40.00	0.891

When we compared the groups in terms of the mean ABG values, we found a statistically significant difference in terms of the mean initial SaO_2_ (p < 0.001) and initial PaO_2_ (p < 0.001) (Table 3).

**Table 3:** Comparison of the average of first blood gas values according to the PaO2 values of the patients a = Kruskal-Wallis H test; Med. = median; Min = minimum; Max = maximum; PaO_2_ = arterial partial pressure of oxygen (mmHg); PaCO_2 _= arterial partial pressure of carbon dioxide (mmHg); BE = base excess; SaO_2 _= oxygen saturation of the arterial blood.

	PaO_2_												
	0-120 mmHg (n = 11)				120-200 mmHg (n = 88)				>200 mmHg (n = 20)				
	Mean	Med.	Min	Max	Mean	Med.	Min	Max	Mean	Med.	Min	Max	p^a^
Initial pH	7.40	7.41	7.18	7.50	7.38	7.39	7.17	7.61	7.36	7.37	7.14	7.50	0.221
Initial SaO_2_	96.97	97.20	94.60	99.10	97.08	97.00	91.60	99.50	98.71	99.00	97.00	99.90	<0.001
Initial PaO_2_	111.85	114.00	100.00	120.20	152.22	150.00	121.00	194.90	233.70	226.05	202.00	287.00	<0.001
Initial PaCO_2_	39.80	37.30	24.00	51.00	41.50	39.95	20.00	65.90	40.14	40.45	28.20	57.60	0.660
Initial lactate	1.49	1.46	0.89	2.39	2.13	1.75	0.47	6.50	2.67	2.19	0.76	11.26	0.057
Initial BE	-0.77	-0.50	-9.50	5.40	-0.71	-0.75	-10.50	10.00	-2.42	-1.65	-14.00	8.30	0.408
Initial HCO_3_	21.87	23.40	2.50	30.00	24.13	24.00	16.00	34.00	22.58	22.00	14.20	33.40	0.278

We found a statistically significant difference (Table 4) when the mean ABG values after treatment were compared: pH (p = 0.010), SaO_2_ (p = 0.008), PaO_2_ (p < 0.001), PaCO_2_ (p = 0.006), lactate (p = 0.015), and the mean of BE (p = 0.035) and HCO3 (p = 0.024).

**Table 4:** Comparison of the post-treatment blood gas values according to the PaO2 values of the patients a = Kruskal-Wallis H test; Med. = median; Min = minimum; Max = maximum; PaO_2_ = arterial partial pressure of oxygen (mmHg); PaCO_2_ = arterial partial pressure of carbon dioxide (mmHg); BE = base excess; SaO_2_ = oxygen saturation of the arterial blood.

	PaO_2_												
	0-120 (1) mmHg (n = 11)				120-200 (2) mmHg (n = 88)				>200 (3) mmHg (n = 20)				
	Mean	Med.	Min	Max	Mean	Med.	Min	Max	Mean	Med.	Min	Max	p^a^
Last pH	7.47	7.48	7.39	7.52	7.41	7.42	7.05	7.54	7.37	7.39	7.22	7.56	0.010
Last SaO_2_	96.50	96.95	92.90	98.30	96.62	96.90	92.90	99.40	98.12	98.60	97.00	99.40	0.008
Last PaO_2_	109.38	109.80	93.90	119.00	150.29	146.70	120.70	195.00	222.16	217.20	204.00	257.00	<0.001
Last PaCO_2_	34.42	35.70	24.00	50.00	41.83	41.00	25.00	80.00	45.00	42.00	16.00	100.00	0.006
Last lactate	1.11	0.91	0.77	2.24	1.38	1.16	-1.05	6.71	1.99	1.40	1.19	6.00	0.015
Last BE	1.28	1.35	-3.40	6.10	2.06	2.05	-20.00	46.00	-2.56	-0.90	-8.80	5.20	0.035
Last HCO_3_	24.63	24.20	19.40	30.00	26.26	26.20	14.50	32.40	22.39	22.90	14.00	29.00	0.024

There was no statistically significant difference between the groups in terms of hospitalization duration (p = 0.373) and length of ICU stay (p = 0.442) (Table 5).

**Table 5:** Comparison of the average of hospital and intensive care hospitalization days according to the initial PaO2 values of the patients a = Kruskal-Wallis H test; Min = minimum; Max = maximum; PaO_2_ = arterial partial pressure of oxygen (mmHg).

	PaO_2_												
	0-120 mmHg (n = 11)				120-200 mmHg (n = 88)				>200 mmHg (n = 20)				
	Mean	Median	Min	Max	Mean	Median	Min	Max	Mean	Median	Min	Max	p^a^
Hospital stay	29.18	10.00	8.00	128.00	18.77	12.00	2.00	147.00	15.40	8.00	2.00	65.00	0.373
ICU stay	24.45	8.00	2.00	124.00	11.57	5.00	1.00	90.00	12.85	6.00	1.00	50.00	0.442

A statistically significant difference was found between groups (groups 1, 2, and 3) in terms of mortality (p < 0.001) and reoperation (p = 0.001) rates. There was no mortality in any of the cases in group 1, only three (27.0%) patients had reoperation, 15 (17%) patients died, and two (2.2%) had re-operation in group 2, and in Group 3, 13 (65.0%) of the patients died and none of them had reoperation (Table 6).

**Table 6:** Comparison of mortality and reoperation rates according to PaO2 values of patients PaO_2_ = arterial partial pressure of oxygen (mmHg).

		PaO_2_						
		0-120 mmHg (n = 11)		120-200 mmHg (n = 88)		>200 mmHg (n = 20)		p
		n	%	n	%	n	%	
Mortality	Yes	0	0.0	15	17	13	65	<0.001
	No	11	100.0	73	83	7	35	
Reoperation	Yes	3	27.0	2	2.2	0	0.0	0.001
	No	8	73.0	86	97.8	20	100.0	

In patients with head trauma in the ICU, the mortality rate was 28 (23.5%). When cases with mortality and without mortality were compared, GCS (p < 0.001), APACHE (p < 0.001), initial SaO_2_ (p = 0.002), extubation day (p < 0.001), last pH (p < 0.001), last PaO_2_ (p < 0.001), last PaCO_2_ (p = 0.015), last lactate (p < 0.001), last BE (p < 0.001), last HCO3 (p < 0.001), and ICU admission (p = 0.038) rates were found to be significantly different (Table 7).

**Table 7:** Comparison of clinical characteristics of the patients in the absence or presence of mortality GCS = Glasgow Coma Scale; APACHE = Acute Physiology and Chronic Health Evaluation score; a = Kruskal-Wallis H test; Med. = median; Min = minimum; Max = maximum; PaO_2_ = arterial partial pressure of oxygen (mmHg); PaCO_2_ = arterial partial pressure of carbon dioxide (mmHg); BE = base excess; SaO_2_ = oxygen saturation of the arterial blood; HCO3 = bicarbonate.

	Mortality								
	Yes (n = 22)				No (n = 97)				
	Mean	Med.	Min	Max	Mean	Med.	Min	Max	p^a^
GCS	5.86	5.00	3.00	11.00	8.91	10.00	3.00	15.00	<0.001
APACHE	27.00	25.50	13.00	45.00	16.45	16.00	10.00	29.00	<0.001
Initial pH	7.38	7.38	7.21	7.61	7.38	7.39	7.14	7.53	0.750
Initial SaO_2_	98.00	98.10	91.60	99.50	97.19	97.00	92.10	99.90	0.002
Initial PaO_2_	171.84	160.50	105.00	258.10	159.99	151.30	100.00	287.00	0.311
Initial PaCO_2_	40.42	40.45	20.00	58.00	41.27	39.90	24.00	65.90	0.787
Initial lactate	2.28	2.21	0.76	5.57	2.13	1.71	0.47	11.26	0.406
Initial BE	-1.81	-1.90	-7.10	4.90	-0.82	-0.50	-14.00	10.00	0.220
Initial HCO_3_	21.97	22.55	2.50	28.80	24.05	24.00	14.20	34.00	0.085
Extubation day	0.00	0.00	0.00	0.00	5.32	2.00	0.00	75.00	<0.001
Last pH	7.31	7.32	7.05	7.56	7.44	7.44	7.33	7.54	<0.001
Last SaO_2_	96.52	97.00	93.40	99.40	96.77	97.00	92.90	99.00	0.810
Last PaO_2_	181.17	182.15	120.70	257.00	144.89	141.00	93.90	232.00	<0.001
Last PaCO_2_	47.77	45.15	16.00	100.00	39.86	39.00	24.00	58.00	0.015
Last lactate	2.29	1.79	0.28	6.71	1.19	1.10	-1.05	3.30	<0.001
Last BE	-3.20	-2.40	-13.00	6.10	2.73	2.30	-20.00	46.00	<0.001
Last HCO_3_	22.61	23.40	14.00	31.40	26.52	26.90	19.40	32.40	<0.001
Hospital stay	18.36	12.00	3.00	128.00	19.35	12.00	2.00	147.00	0.755
ICU stay	16.09	8.50	2.00	124.00	12.27	5.00	1.00	90.00	0.038

However, there was no statistically significant difference between the two groups in terms of the diagnosis types (p = 0.022), ASA (p = 0.015), mean BMI values, gender distribution (p = 0.109), and comorbidity rates (p = 0.006). In terms of mean age, there was a statistically significant difference (p < 0.001) between the two groups, and the mean age was higher in the group with mortality.

## Discussion

Oxygen (O_2_) therapy is frequently applied as a life-saving treatment in critically ill patients. Generally, hypoxia has been considered harmful up to date, and above-normal arterial O_2_ levels have been allowed during treatment [7].

Reactive oxygen metabolites (ROM) were initially recognized as toxic by-products of aerobic metabolism. Rather than O_2_ itself, ROM shows toxic effects. Since ROM can cause serious harmful side effects, ROM limits the use of O_2_, especially under ischemia/reperfusion conditions. Prolonged resuscitation times after severe trauma may be associated with high PaO_2_. Although these effects are particularly evident during long-term administration, there are retrospective studies suggesting that even short-term hyperoxemia is associated with increased mortality and morbidity [8]. Long-term (≥24 hours) exposure to hyperoxia has been shown to partially reduce mitochondrial respiratory capacity and inhibit complexes I and II [9,10].

Observational and interventional studies evaluating the relationship between hyperoxia and mortality were reviewed. The studies were generally heterogeneous, retrospective, and in large groups. To limit this heterogeneity, we have chosen a more specific group of patients who were hospitalized for head trauma in the ICU.

In our study, we could not specify hyperoxia with a precise value (such as PaO_2_ over 300 mmHg), but we considered the second group PaO_2_ between 120 and 200 mmHg as mild hyperoxic and the third group with a PaO2 > 200 mmHg as hyperoxic. The fraction of inspired oxygen (FiO_2_) values, intubation and extubation times, and mechanical ventilation (MV) settings are not regularly recorded in the epicrisis and files of the patients. Since these patients were admitted from the emergency department or operating room to the ICU while breathing spontaneously, we accepted FiO_2_ values as <50%.

In our study, there was a statistically significant difference in terms of initial PaO_2_ and SaO_2 _values between the groups. But this result is not clinically significant, as we had already determined the groups according to their PaO_2_ values. Nevertheless, when we compared ABG samples taken after the treatment, we also found a statistically significant difference between the groups in terms of pH, PaO_2_, PaCO_2_, SaO_2_, lactate, BE, and HCO3 values.

The fact that these values are higher in the mortality group is clinically significant because especially lactate and BE are among the biomarkers that show tissue oxygenation and perfusion in critically ill patients [11].

The role of hyperoxia in traumatic brain injury (TBI) is still controversial. In the management of TBI, from a pathophysiological point of view, it is widely believed that hyperoxia-induced vasoconstriction theoretically allows the lowering of intracranial pressure (ICP) and improves cerebral perfusion pressure. Contrary to normobaric hyperoxia, cerebral blood flow is increased in hyperbaric hyperoxia [12]. With ICP measurement in patients with head trauma, the effect of hyperoxia on cerebral perfusion pressure can be made significant with a prospective study. As a result, it can be demonstrated whether hyperoxia will have a different effect on the cerebral tissue despite the damage to the pulmonary, cellular, and some organs.

In a study by Rockswold et al., after combining hyperbaric oxygen therapy with normobaric hyperoxia, it was observed that GCS improved in nine of 22 patients in the long term and mortality decreased within six months [13]. However, when normobaric hyperoxia was applied alone, no significant results were found [14]. Hyperbaric oxygen therapy is not applied in our hospital. However, in our study, in which we can only mention normobaric hyperoxia, GCS was found to be lower in hyperoxic groups 2 and 3, and a statistically significant difference was found in terms of GCS and APACHE scores between groups with and without mortality. GCS was found to be lower in the mortality group. This is a clinically expected result.

In a large retrospective cohort analysis conducted by Davis et al. in 2009 including 3,420 patients, it was found that hypoxemia (PaO_2 _< 100 mmHg) increases mortality in TBI [15]. Whereas, in our study, we found that mortality decreased below 110 mmHg. Partial pressures between 250 and 486 mmHg have been associated with improved all-cause survival. Studies have shown that normoventilation and mild hyperoxemia can be considered ideal in patients under MV therapy [16]. While hypercapnia increases ICP with cerebral vasodilation, hypocapnia (PaCO2 < 35 mmHg) may cause secondary brain damage with cerebral vasodilation. In this study, PaO_2_ = 250 mmHg was considered hyperoxic, and PaO2 > 400 mmHg was considered extremely hyperoxic. In a very comprehensive study, they separated the patients in the ICU according to their diagnoses, and in the TBI group, significant results were found in mortality. In our study, the patient group with head trauma was selected to narrow this heterogeneity. In patients with TBI, the effects of hypo and hypercapnia on brain damage and mortality in those on MV should not be ignored.

In a prospective, single-center observational study conducted by Jeon et al. involving 252 patients, it was reported that hyperoxemia (defined as PaO_2_ > 173 mmHg) was associated with delayed cerebral ischemia and neurological deterioration [17]. In our study, we also selected a patient group with TBI. PaO_2_ values were similar and mortality was higher in the mildly and significantly hypoxemic groups. However, no statistically significant difference was found in terms of hospital and ICU length of stay.

In contrast, a retrospective analysis of 2,643 adults in ICUs in Australia and New Zealand failed to show a significant relationship between mortality and PaO_2_ values in the first 24 hours in patients on MV with ischemic stroke [18].

In a multicenter cohort study of 6,326 patients, hyperoxemia (PaO_2_ > 300 mmHg) was associated with higher mortality than normoxemia. A secondary analysis of 4,459 patients showed a linear relationship between increases in PaO_2_ and increased risk of mortality [19].

In the only randomized controlled trial comparing 14 patients in each group, an increase in neuron-specific enolase levels at 24 hours was shown in the hyperoxic group [20]. Recently, in two more interesting retrospective cohort analyses, severe hyperoxia has been associated with reduced survival and poor neurological outcome [21,22].

In a retrospective analysis of 36,307 patients, the correlation between in-hospital mortality and arterial PaO_2_ measurements during the first 24 hours of ICU hospitalization was shown to be the lowest between 110 and 150 mmHg values, while the mortality was increased below 67 mmHg and above 225 mmHg [23]. In our study, similar to this study, the increase in mortality in the second and third groups with PaO_2_ = 120-200 and >200 was parallel to hyperoxemia. In ICU patients on MV, the FiO_2_ value does not exceed 50%, except in conditions such as lung pathology, cardiopulmonary resuscitation, or acute respiratory distress syndrome. In this case, most of the patients were concentrated in the group with PaO_2_ = 120-200 mmHg.

Janz et al. evaluated three multicenter and three single-center retrospective cohort studies with patients who were resuscitated [20]. Different PaO_2_ measurements have been used to define hyperoxia exposure, generally using a PaO_2_ cut-off value of 300 mmHg. The relationship between mortality and PaO_2_ was evaluated by multivariate regression analysis. To limit heterogeneity between studies, for both the definition and analysis of hyperoxia, they grouped patients based on the 300 mmHg PaO_2_ threshold by stratifying patients between hyperoxic and non-hyperoxic groups and subtracting the number of survivors/non-survivors in the two groups.

In two multicenter retrospective cohort studies evaluating the relationship between in-hospital mortality and exposure to hyperoxia in the first 24 hours of ICU stay in stroke patients, Rincon et al. defined the patients with an initial PaO_2_ of 300 mmHg and above as those exposed to hyperoxia [24].

Helmerhorst et al. conducted a meta-analysis and meta-regression analysis of cohort studies published between 2008 and 2015 to demonstrate the deleterious effects of hyperoxia in critically ill patients [25]. Publications assessing the effect of arterial hyperoxia on outcomes in critically ill adults (≥18 years) admitted to critical care units were eligible. Twenty-four studies were included, of which five studies were only for a subset of the analyses. Nineteen studies were pooled for meta-analyses and showed that arterial hyperoxia during admission increases hospital mortality. Studies on patients with chronic obstructive pulmonary disease, extracorporeal life support or hyperbaric oxygen therapy, and animal studies were excluded. The primary goal was stated as an assessment of in-hospital mortality. Functional outcome measures were diverse and generally showed a more favorable outcome for normoxia. Although this study showed similarities with our study, we think that the reason for the results they found is heterogeneity and group size.

After prolonged and severe exposure to hypoxia or hyperoxia, the physiological reserve is depleted and the defense mechanism is collapsed, resulting in tissue damage and neuronal damage. Because of its low regenerative potential, hypoxia-inducible factor 1-alpha (HIF-1α) is elevated as a marker of brain damage [26]. There is not enough study about the brain damage caused by hyperoxia.

Benderro et al. showed that the expression of angiogenic vascular endothelial growth factor (VEGF) decreased in patients receiving 50% FiO_2_ oxygen therapy for 21 days, resulting in impaired capillary passage [27]. Unfortunately, data about brain injury are lacking in this study.

During the delivery of oxygen at different FiO_2_ levels, the brain increases the expression of HIF-1α to protect its functions. Especially with the increase of VEGF, mechanisms that increase cerebral blood flow by vascular remodeling can be triggered [28]. In rats exposed to FiO_2_ = 0.5 for three weeks, brain capillary density decreased with decreased levels of VEGF protein and VEGF mRNA, despite increased HIF-1α and hypoxia-inducible factor 2-alpha (HIF-2α) proteins. Short-term hyperbaric hyperoxia reduces cerebral blood flow by causing nitric oxide (NO) reduction and consequent vasoconstriction due to the increased production of superoxide anions that inactivate NO. There is increasing evidence that O_2_ regulates the fate of central nervous system precursors through the activation of neural stem cells (NSCs), whose proliferation and multipotential are enhanced by mild hypoxia.

In a study conducted by De Jonge et al. in the Netherlands, the mean PaO_2_ and FiO_2_ values in the first 24 hours were taken from the ABGs of MV-dependent patients who were hospitalized in the ICU and PaO_2 _> 120 mmHg was accepted as hyperoxemia [23]. In the study, a linear relationship was found between hospital mortality and FiO_2_ values. Very low and very high PaO_2_ values have been associated with high mortality. In this study, PaO_2_ values equivalent to our study were accepted as hyperoxia and similar results were obtained.

## Conclusions

In our study, patients with head trauma followed in the ICU were divided into groups according to the highest initial PaO_2_ values in ABG samples and their mortality was compared. A significant difference was found between the hyperoxic and normoxic groups in terms of mortality. More comprehensive studies are needed to understand the short and long-term effects of oxygen therapy better and to fully evaluate the relationship of the oxidant and antioxidant system with hyperoxia. In addition to this, it is thought that it will be beneficial in terms of reducing mortality, length of hospital stay, and financial and physical losses, especially in critically ill patients.

Since our study is retrospective, it was conducted based on the data in the patient records. However, data about the intubation and extubation times and mechanical ventilatory settings were recorded incompletely in the files. In addition, the way of oxygen therapy administration (nasal, mask with reservoir, high flow, etc.), the flow rates, percentages, and duration were not recorded. All of these factors limited the ability of our study to show the precise effect of hyperoxygenation on mortality and morbidity. So we think that a prospective study, in which these data will be included completely, will be more effective in this regard.
